# Cyclooctyne End‐Functionalized Poly(morpholine‐2,5‐dione)s

**DOI:** 10.1002/marc.202400705

**Published:** 2024-11-29

**Authors:** Johanna Schreiber, Natalie E. Göppert, Leanne M. Stafast, Christine Weber, Ulrich S. Schubert

**Affiliations:** ^1^ Laboratory of Organic and Macromolecular Chemistry (IOMC) Friedrich Schiller University Jena Humboldtstr. 10 07743 Jena Germany; ^2^ Jena Center for Soft Matter (JCSM) Friedrich Schiller University Jena Philosophenweg 7 07743 Jena Germany

**Keywords:** click chemistry, morpholine‐2,5‐dione, poly(ester amide), polydepsipeptide, ring‐opening polymerization, strain‐promoted azide‐alkyne cycloaddition (SPAAC)

## Abstract

The cyclooctyne‐functionalized alcohol (1*R*,8*S*,9*S*)‐bicyclo‐[6.1.0]non‐4‐yn‐9‐ylmethanol (BCN‐OH) is applied as initiator for the organo‐catalyzed ring‐opening polymerization (ROP) of morpholine‐2,5‐diones based on the l‐amino acids valine, isoleucine, and phenylalanine. The ROP is catalyzed by a binary system of 1,8‐diazabicyclo[5.4.0]undec‐7‐ene (DBU) and 1‐(3,5‐bis(trifluoromethyl)phenyl)‐3‐cyclohexylthiourea (TU) applying a feed ratio of [M]/[I]/[DBU]/[TU] of 100/1/1/10. Kinetic studies reveal that BCN‐OH is capable to initiate the polymerization of morpholine‐2,5‐diones, which proceed in a controlled manner until monomer conversions of 80%. Characterization by means of ^1^H NMR spectroscopy, size exclusion chromatography (SEC), and matrix‐assisted laser desorption/ionization‐time of flight‐mass spectrometry confirm the covalent attachment of the cyclooctyne moiety as α‐end group of the poly(morpholine‐2,5‐dione)s with maximum dispersities of 1.25. As a proof of concept, a vitamin A end‐functionalized poly(2‐ethyl‐2‐oxazoline) is coupled to a poly(ester amide) by strain‐promoted azide‐alkyne cycloaddition. Characterization of the block copolymer by SEC and DOSY NMR spectroscopy confirm the successful attachment of the two building blocks. The versatile cyclooctyne moiety shall facilitate a metal‐free attachment of other polymer blocks, targeting ligands or dyes at the α‐end group of well‐defined poly(morpholine‐2,5‐dione)s. In consequence, the approach provides access to a new generation of functionalized poly(ester amide)s, which can be customized for specific needs.

## Graphical abstract

This is the first report of a successful initiation of the ring‐opening polymerization of morpholine‐2,5‐diones based on l‐valine, l‐isoleucine, and l‐phenylalanine using a cyclooctyne derivative as an initiator. The post‐polymerization modification via strain‐promoted azide‐alkyne cycloaddition is demonstrated by coupling a poly(ester amide) with a hydrophilic and vitamin A end‐functionalized “stealth” polymer.

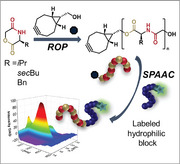

## 1 Introduction

Poly(ester amide)s are in the focus of research as alternatives to polyesters for biomedical applications.^[^ ^1^, ^2^, ^3^ ^]^ In addition to the ester moieties, these biodegradable and biocompatible polymers also feature more stable amide bonds in the backbone. This provides more parameters to tailor the polymers to specific applications.^[^ ^2^, ^3^ ^]^ For example, an advanced adjustment of the degradation of the corresponding drug delivery systems, which may occur under acidic, basic or enzymatic conditions, is possible.^[^ ^1^, ^4^ ^]^ Furthermore, the hydrophobicity and, thus, the interactions between polymer and drug can be influenced by the composition of the ester and amide moieties, but also by the choice of suitable monomer substituents.^[^ ^3^, ^4^ ^]^ By incorporating aromatic substituents, an additional attractive interaction with drugs, which also often contain conjugated systems, can be introduced via π–π stacking.^[^ ^5^ ^]^

The ring‐opening polymerization (ROP) of morpholine‐2,5‐diones represents one option to obtain poly(ester amide)s.^[^ ^1^, ^6^, ^7^ ^]^ As these monomers are not commercially available, the synthesis of the respective compounds, for example, from naturally occurring l‐amino acids, is necessary.^[^ ^1^, ^5^, ^8^, ^9^, ^10^ ^]^ The homopolymerization of a morpholine‐2,5‐dione by ROP was first described by Helder et al.^[^ ^6^ ^]^ in 1985. Here, 6‐methylmorpholine‐2,5‐dione was polymerized in bulk in the presence of tin(II)‐ethylhexanoate as catalyst. Since then, there have been many developments in this field.^[^ ^1^, ^11^, ^12^, ^13^ ^]^ In addition to metal‐based ROP, a polymerization using enzyme‐catalyzed approaches has been mainly described by Feng et al.^[^ ^12^, ^14^, ^15^, ^16^, ^17^ ^]^

Organo‐based ROP of morpholine‐2,5‐diones was pioneered by Hedrick and coworkers in 2005, describing the use of *N*‐heterocyclic carbenes and thiourea for catalysis.^[^ ^18^, ^19^ ^]^ Since then, 1,5,7‐triazabicyclo[4.4.0]dec‐5‐ene (TBD) and 1,8‐diazabicyclo[5.4.0]undec‐7‐ene (DBU) have come into focus as catalysts.^[^ ^20^, ^21^ ^]^ Moreover, narrower dispersities and higher monomer conversions have been achieved in combination with thiourea derivatives as cocatalysts.^[^ ^1^, ^5^, ^10^, ^22^, ^23^ ^]^ The possibility to perform the polymerization at room temperature using such catalyst systems might enable the use of temperature‐sensitive reagents during the reaction.

Similar to metal‐catalyzed ROP, primary alcohols such as benzyl alcohol act as initiators in organo‐catalyzed ROP, thereby defining the α‐end group of the resulting poly(ester amide). Although this has been exploited for the formation of block copolymers through utilization of macroinitiators,^[^ ^5^, ^20^ ^]^ the possibility to attach end groups with added value via this straightforward route has, to the best of our knowledge, not yet been explored.

In particular, cyclooctyne derivatives seem highly promising since they enable the subsequent coupling of various building blocks carrying an azide functionality via strain‐promoted azide‐alkyne cycloaddition (SPAAC). Such a strategy may, for example, be utilized for attachment of labels^[^ ^24^, ^25^ ^]^ or other polymeric building blocks exhibiting a “stealth” effect.^[^ ^26^, ^27^ ^]^ In fact, the cyclooctyne derivative (1*R*,8*S*,9*S*)‐bicyclo‐[6.1.0]non‐4‐yn‐9‐ylmethanol (BCN‐OH) has been successfully used for the initiation of the organo‐based ROP of lactide and ε‐caprolactone.^[^ ^28^, ^29^ ^]^

**Scheme 1:**
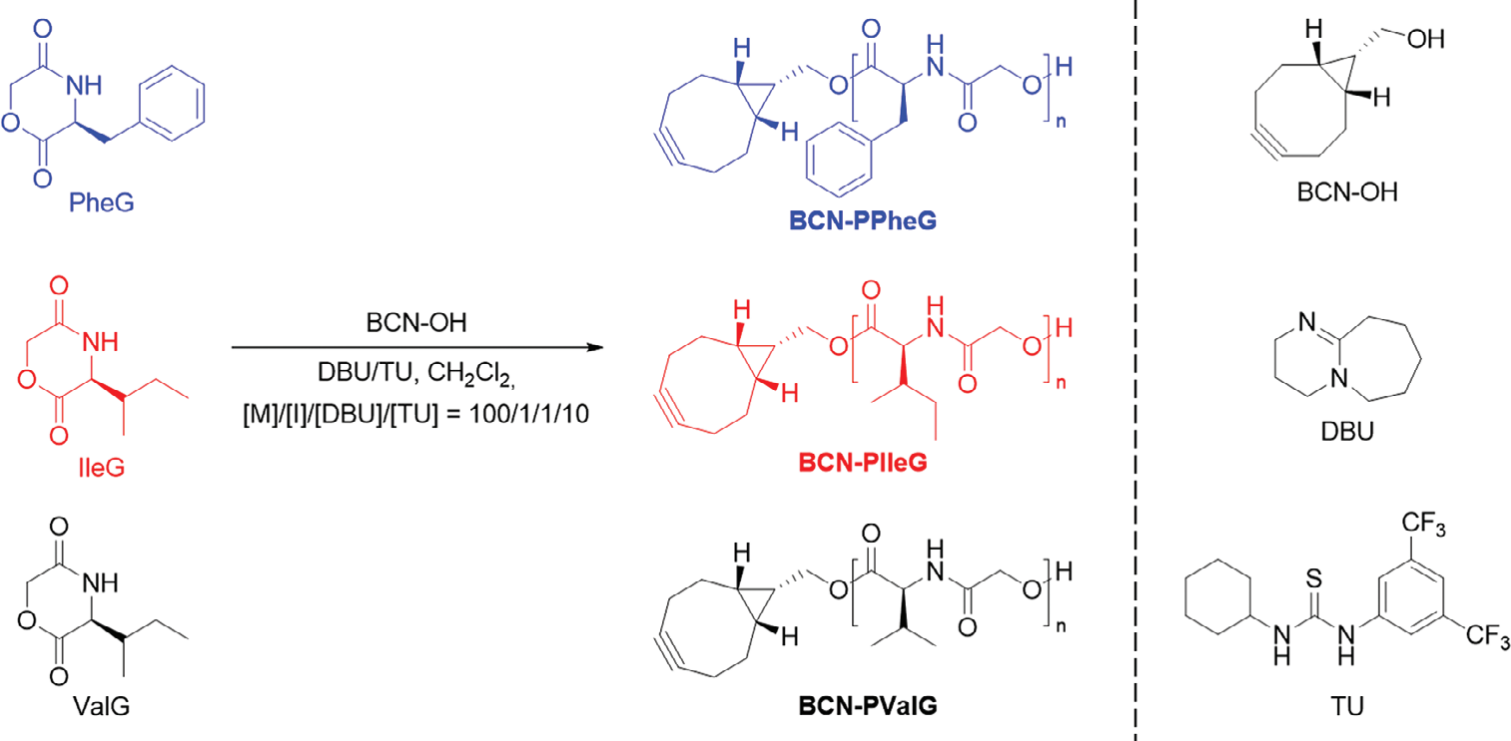
Schematic representation of the synthesis route toward the polymers **BCN‐PPheG**, **BCN‐PIleG**, and **BCN‐PValG** using the corresponding monomers PheG, IleG, and ValG.

Herein, we describe the organo‐catalyzed ROP of morpholine‐2,5‐diones based on the natural α‐amino acids l‐valine (ValG), l‐isoleucine (IleG), and l‐phenylalanine (PheG) initiated by BCN‐OH (**Scheme** **1**). Initially, suitable ROP conditions were established using DBU in combination with 1‐(3,5‐bis(trifluoromethyl)phenyl)‐3‐cyclohexylthiourea (TU) as catalytic system at room temperature. The α‐end group fidelity was investigated by mass spectrometry. As a proof of principle, an azide end‐functionalized poly(2‐ethyl‐2‐oxazoline) (PEtOx) additionally carrying retinoic acid amide was conjugated to a cyclooctyne functionalized poly(ester amide) via SPAAC.

## 2 Results and Discussion

The monomers for the polymerizations described below were synthesized following the commonly used routes.^[^ ^5^, ^30^ ^]^ Therefore, the corresponding l‐amino acid was reacted with chloroacetyl chloride in tetrahydrofuran in the presence of triethylamine. The obtained precursor subsequently underwent a cyclization reaction at high dilution in *N,N*‐dimethylformamide using NaHCO_3_ as base. A detailed description of the procedure is available in the Supporting Information.

### 2.1 Kinetic Studies

Similar to reaction conditions typically used for the DBU/TU‐catalyzed ROP of morpholine‐2,5‐diones,^[^ ^5^, ^10^, ^22^, ^23^, ^30^ ^]^ the reactions were carried out at room temperature in CH_2_Cl_2_ at a [M]/[I]/[DBU]/[TU] ratio of 100/1/1/10.

Kinetic studies were performed to investigate the polymerization of PheG, IleG, and ValG using BCN‐OH as initiator (Scheme 1). Samples taken from the reaction solutions at specific time intervals were analyzed by means of ^1^H NMR spectroscopy and size exclusion chromatography (SEC) to determine the monomer conversions and the number average of the molar masses (*M*_n_).

First, an initial monomer concentration ([M]_0_) of 0.5 mol L^−1^ was set in the ROP of PheG, i.e., the same concentration used in the adapted polymerization procedure by Göppert et al.^[^ ^5^ ^]^ However, the formation of a high molar mass shoulder of the distribution was observed in the SEC elugrams already at a monomer conversion of 42% (Figure S2, Supporting Information). As evident from the increase of the *M*_n_ values with the monomer conversions with a flattening slope, molar mass control could not be achieved at these reaction conditions. Thereby, the dispersities increased up to 1.40 during the considered reaction time frame. These data point toward the presence of a significant amount of intermolecular chain transfer reactions. Reducing the monomer concentration or increasing the amount of TU may result in an improvement of reaction control.^[^ ^5^ ^]^ This is reasonable as a lower monomer and polymer concentration would decrease the probability of intermolecular transesterifications. At a [M]_0_ of 0.3 mol L^−1^, unimodal molar mass distributions were maintained until conversions around 50%, and dispersities remained below 1.3 even at monomer conversions above 80% (Figure S3, Supporting Information). Accordingly, these polymerization conditions were also applied for the ROP of IleG and ValG.

**Figure 1:**
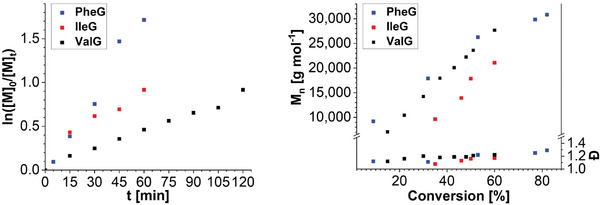
Semilogarithmic kinetic plots (left) and plots of *M*_n_ and Ð determined by SEC against the monomer conversion (right) for the BCN‐OH‐initiated polymerizations of PheG, IleG, and ValG ([M]/[I]/[DBU]/[TU] = 100/1/1/10, [M]_0_ = 0.3 mol L^−1^, room temperature).

**Figure** **1** displays the kinetic plots based on the obtained data from ^1^H NMR spectroscopy and SEC. In particular, the ROP of PheG and ValG followed a linear trend in the semi‐logarithmic kinetic plot. The polymerization of PheG proceeded significantly faster than that of ValG, as evidenced by the increased slope. In contrast, a significant decrease of the slope in the semilogarithmic plot for the ROP of IleG was observed in an early stage of the reaction. Despite that, the molar mass of the **BCN‐PIleG** increased in a linear fashion with the monomer conversion, which was also the case for **BCN‐PValG**. In case of **BCN‐PPheG**, the slope flattened slightly above conversions of around 50%, but molar mass control was significantly improved compared to the ROP conducted at a [M]_0_ of 0.5 mol L^−1^.

The SEC elugrams of the kinetic samples obtained from the polymerization of IleG displayed a slight low molar mass tailing (Figure S3, Supporting Information), which may indicate that chain terminations or intramolecular chain transfers could not be fully avoided, although dispersities remained below 1.16. Unimodal and narrow molar mass distributions with dispersities below 1.21 were found throughout the ROP of ValG. A slight high molar mass shoulder only occurred at conversions above 60%.

In general, chain transfer reactions through transesterification occurred only towards the end of the polymerizations, which suggested that the desired cyclooctyne end group would be present at the majority of polymer chains if the ROP were terminated at appropriate reaction times.

### 2.2 Synthesis of Cyclooctyne End‐Functionalized Poly(ester amide)s

The syntheses of **BCN‐PPheG**, **BCN‐PIleG**, and **BCN‐PValG** were performed using the reaction conditions described in the previous section. The ROP of ValG and IleG were driven to conversions around 80%, whereas the polymerization of PheG was terminated at a conversion of 29% (**Table** **1**). The expected degrees of polymerization (DP_theo_) correspond to molar masses between 6 and 14 kg mol^−1^. The molar masses determined by SEC significantly exceed those values, hinting toward a large hydrodynamic volume of all poly(ester amide)s in the eluent. In line with the kinetic studies, dispersity values around 1.2 were determined. Whereas the molar mass distribution of **BCN‐PIleG** revealed a high molar mass shoulder, monomodal distributions were detected for **BCN‐PPheG** and **BCN‐PValG** (Figure S5, Supporting Information).

**Table 1:** Selected characterization data of the poly(ester amide)s and the block copolymer.

	Conv.^a)^ [%]	DP_theo_ ^b)^	*M*_n,theo_ ^b)^ [g mol^−1^]	*M*_n_ ^c)^ [g mol^−1^]	Ɖ^c)^
**BCN‐PValG**	83	83	13 200	35 800	1.19
**BCN‐PIleG**	81	81	14 000	25 800	1.25
**BCN‐PPheG**	29	29	6 100	20 600	1.19
**Ret‐PEtOx‐*b*‐PValG**	–	19/83	15 500	35 800	1.23

^a)^ Determined from the ^1^H NMR spectra of the reaction solutions;

^b)^ Calculated from [M]_0_/[I] and conversion;

^c)^ Determined by SEC (DMAc, 0.21% w/w LiCl; RID; PS calibration).

Due to the rather high DP_theo_ values and overlapping signals of the repeating units, end group signals derived from the cyclooctyne end group were difficult to detect and to assign in the ^1^H NMR spectra, which, nevertheless, confirm the identities of the repeating units (compare assignment in Figure S6, Supporting Information).

**Figure 2:**
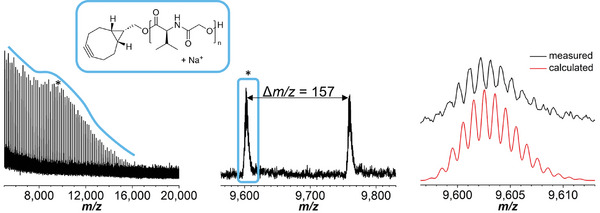
MALDI‐TOF MS analysis of **BCN‐PValG**. Left: Full mass spectrum (DCTB, NaTFA) of **BCN‐PValG** with highlighted distribution of the detected species. Middle: Zoom into the spectrum. Right: Overlay of the measured and the calculated isotopic pattern of the detected species.

The α‐end group fidelity was hence investigated by means of matrix‐assisted laser desorption/ionization‐time of flight‐mass spectrometry (MALDI‐TOF MS). **Figure** **2** depicts the MALDI mass spectrum of **BCN‐PValG**. The spectra of **BCN‐PIleG** and **BCN‐PPheG** are shown in the Supporting Information (Figures S7 and S8, Supporting Information). In all spectra, the main *m/z* distribution was assigned to the respective polymer with the desired α‐end group, as evident from the overlay of the measured and calculated isotopic patterns. For **BCN‐PValG**, the sodium adducts represented the only species detected until *m/z* values of 16 000. The occurrence of molar mass discrimination effects at such high *m/z* values is not surprising. Nevertheless, non‐fragmented macromolecules could be ionized in a molar mass range that corresponds to *M*_n,theo_. Irrespective of the sample, spectra measured with dithranol as a matrix instead of *trans*‐2‐[3‐(4‐*tert*‐butylphenyl)‐2‐methyl‐2‐propenylidene]malononitrile (DCTB) revealed more *m/z* series. In the former, macromolecules with carboxylic acid or carboxylate end groups were detected frequently. Such polymer chains could theoretically be formed by initiation of the ROP with traces of water. However, their high abundance, particularly in the lower *m/z* regions of the spectra points toward the formation of those species through fragmentation. Cyclic species indicating intramolecular chain transfer reactions occurring during the ROP were only found in the spectra of **BCN‐PPheG**. In this case, transesterification reactions were anticipated based on the results of the kinetic studies as described above.

In summary, the analytical data demonstrate a successful synthesis of **BCN‐PPheG**, **BCN‐PIleG** as well as **BCN‐PValG** as the presence of the desired cyclooctyne moiety was confirmed.

### 2.3 SPAAC Reaction

**Scheme 2:**
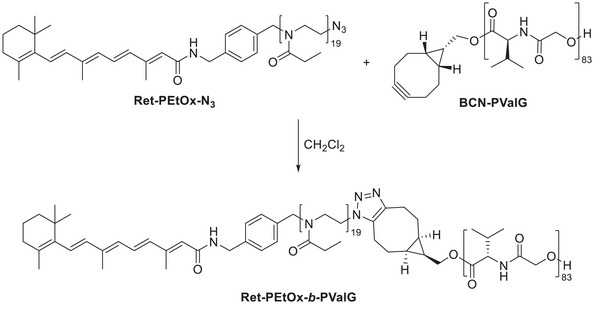
Schematic representation of the SPAAC of **BCN‐PValG** and **Ret‐PEtOx‐N_3_** to produce an amphiphilic block copolymer functionalized with a biologically active molecule.

To demonstrate the accessibility of the cyclooctyne moiety of the synthesized poly(morpholine‐2,5‐dione)s during a SPAAC, **BCN‐PValG** was reacted with **Ret‐PEtOx‐N_3_** in CH_2_Cl_2_ at room temperature (**Scheme** **2**). The azide‐functionalized building block is based on PEtOx as a hydrophilic polymer that exhibits “stealth” properties and vitamin A as a targeting ligand for hepatic stellate cells.^[^ ^27^ ^]^

Figure S9 (Supporting Information) displays an overlay of the ^1^H NMR spectra of **Ret‐PEtOx‐*b*‐PValG** and the two polymeric educts. Signals assigned to both building blocks were observed in the spectrum of the obtained block copolymer. Those appeared at the same diffusion coefficient in the diffusion‐ordered NMR spectrum (DOSY NMR, Figures S10 and S11, Supporting Information), which indicates a successful coupling of the polymers.

**Figure 3:**
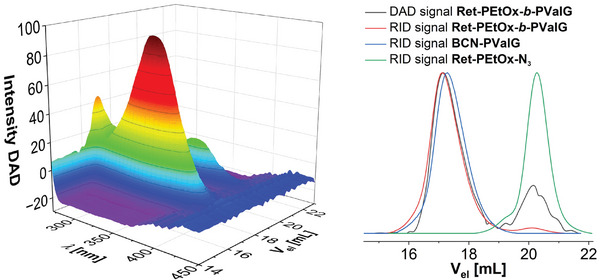
Left: 3D spectrum of the SEC measurement (DMAc, 0.21% w/w LiCl; RID; DAD) of **Ret‐PEtOx‐*b*‐PValG** using DAD. Right: Overlay of the elugrams of **Ret‐PEtOx‐*b*‐PValG** (red), **BCN‐PValG** (blue), and **Ret‐PEtOx‐N_3_** (green) recorded with the RID and the elugram of **Ret‐PEtOx‐*b*‐PValG** recorded with the DAD (351 nm, gray).

To further confirm the covalent attachment of the two blocks, a SEC measurement was carried out with a refractive index detector (RID) and an additional diode array detector (DAD, **Figure** **3**). The block copolymer eluted at slightly lower retention volumes compared to that of **BCN‐PValG**. This is in line with the large hydrodynamic volume of the poly(ester amide) on the SEC system and the comparably low molar mass of **Ret‐PEtOx‐*b*‐PValG** (DP = 19).

The DAD confirmed the successful attachment of the two building blocks as the block copolymer was detected at wavelengths corresponding to the absorption of the conjugated system of the retinoic acid amide‐based end group. **BCN‐PValG** alone does not absorb light between 300 and 450 nm (Figure S12, Supporting Information). A minor amount of **Ret‐PEtOx‐N_3_** was also visible, which may be a result from PEtOx chains lacking the azide functionality.

## 3 Conclusion

In conclusion, we successfully applied the cyclooctyne‐functionalized alcohol BCN‐OH as initiator for the organo‐catalyzed ROP of the morpholine‐2,5‐diones ValG, IleG, and PheG. Kinetic studies revealed a successful initiation of the polymerization, which was catalyzed by DBU and TU. Characterization of the resulting polymers **BCN‐PPheG**, **BCN‐PIleG**, and **BCN‐PValG** by means of ^1^H NMR spectroscopy, SEC and, in particular, MALDI‐TOF MS confirmed the preservation and covalent attachment of the cyclooctyne moiety as α‐end group.

As an exemplary hydrophilic polymer featuring a targeting ligand, **Ret‐PEtOx‐N_3_** was successfully coupled to **BCN‐PValG** as a hydrophobic and degradable building block.

Although the ROP of, in particular, PheG might be further optimized in the future, the versatile cyclooctyne moiety enables a metal‐free attachment of, for example, hydrophilic polymer blocks exhibiting a “stealth” effect, targeting ligands, or dyes at the α‐end group of well‐defined poly(ester amide)s. In consequence, the approach provides access to a new generation of functionalized poly(ester amide)s, which can be customized for the specific needs of various drug delivery systems.

## Conflict of Interest

The authors declare no conflict of interest.

## Supporting information



Supporting Information

## Data Availability

The data that support the findings of this study are available from the corresponding author upon reasonable request.

## References

[1] M. Dirauf , I. Muljajew , C. Weber , U. S. Schubert , Prog. Polym. Sci. 2022, 129, 101547.

[2] M. Winnacker , B. Rieger , Polym. Chem. 2016, 7, 7039.

[3] A. Rodríguez‐Galán , L. Franco , J. Puiggalí , in Biodegradable Polymers: Processing, Degradation and Applications (Ed: G. P. Felton ), Nova Science Publishers, New York 2011.

[4] K. Ghosal , M. S. Latha , S. Thomas , Eur. Polym. J. 2014, 60, 58.

[5] N. E. Göppert , M. Dirauf , P. Liebing , C. Weber , U. S. Schubert , Macromol. Rapid Commun. 2022, 44, e2200651. 36413677 10.1002/marc.202200651

[6] J. Helder , F. E. Kohn , S. Sato , J. W. van den Berg , J. Feijen , Makromol. Chem., Rapid Commun. 1985, 6, 9.

[7] P. J. A. in't Veld , P. J. Dijkstra , J. H. van Lochem , J. Feijen , Makromol. Chem. 1990, 191, 1813.

[8] Methoxypolyethylene glycol‐polyester‐amino acid modified disordered copolymer as well as preparation method and application thereof, CN108383999 (A), 2018.

[9] Y. Ohya , H. Yamamoto , K. Nagahama , T. Ouchi , J. Polym. Sci. Part A: Polym. Chem. 2009, 47, 3892.

[10] J. Lian , M. Li , S. Wang , Y. Tao , X. Wang , Macromolecules 2020, 53, 10830.

[11] Y. Feng , D. Klee , H. Höcker , Macromol. Chem. Phys. 2001, 202, 3120.

[12] Y. Feng , J. Lu , M. Behl , A. Lendlein , Macromol. Biosci. 2010, 10, 1008. 20602421 10.1002/mabi.201000076

[13] Y. Feng , D. Klee , H. Höcker , Macromol. Chem. Phys. 1999, 200, 2276.

[14] Y. Feng , J. Knüfermann , D. Klee , H. Höcker , Macromol. Chem. Phys. 1999, 200, 1506.

[15] Y. Feng , J. Knüfermann , D. Klee , H. Höcker , Macromol. Rapid Commun. 1999, 20, 88.

[16] Y. Feng , D. Klee , H. Höcker , Macromol. Biosci. 2001, 1, 66. 10.1002/mabi.200300067 15468252

[17] Y. Feng , D. Klee , H. Keul , H. Höcker , Macromol. Chem. Phys. 2000, 201, 2670.

[18] R. C. Pratt , A. P. Dove , B. G. G. Lohmeijer , D. A. Culkin , R. M. Waymouth , J. L. Hedrick , Polym. Prepr. 2005, 46, 902.

[19] R. C. Pratt , B. G. G. Lohmeijer , A. Mason , R. M. Waymouth , J. L. Hedrick , Polym. Prepr. 2006, 47, 101.

[20] M. Dirauf , A. Erlebach , C. Weber , S. Hoeppener , J. R. Buchheim , M. Sierka , U. S. Schubert , Macromolecules 2020, 53, 3580.

[21] M. Dirauf , D. Bandelli , C. Weber , H. Görls , M. Gottschaldt , U. S. Schubert , Macromol. Rapid Commun. 2018, 39, 1800433. 10.1002/marc.201800433 30091817

[22] Y.‐T. Guo , C. Shi , T.‐Y. Du , X.‐Y. Cheng , F.‐S. Du , Z.‐C. Li , Macromolecules 2022, 55, 4000.

[23] C.‐X. Shi , Y.‐T. Guo , Y.‐H. Wu , Z.‐Y. Li , Y.‐Z. Wang , F.‐S. Du , Z.‐C. Li , Macromolecules 2019, 52, 4260.

[24] J. Gaitzsch , M. Delahaye , A. Poma , F. Du Prez , G. Battaglia , Polym. Chem. 2016, 7, 3046.

[25] N. Melnychuk , P. Ashokkumar , I. O. Aparin , A. S. Klymchenko , ACS Appl. Polym. Mater. 2022, 4, 44.

[26] N. E. Gppert , M. Dirauf , C. Weber , U. S. Schubert , Polym. Chem. 2021, 12, 5426.

[27] L. M. Stafast , N. Engel , H. Görls , C. Weber , U. S. Schubert , Eur. Polym. J. 2023, 184, 111779.

[28] S. A. van den Berg , H. Zuilhof , T. Wennekes , Macromolecules 2016, 49, 2054.

[29] N. E. Goeppert , A. Vollrath , L. M. Stafast , S. Stumpf , B. Schulze , S. Hoeppener , C. Weber , U. S. Schubert , RSC Appl. Polym. 2024, 2, 184.

[30] T. F. Burton , Z. Garisoain , C. Chaix , J. Aassine , E. Virapin , A. Voronova , J. Pinaud , O. Giani , ACS Omega 2024, 9, 28583. 38973935 10.1021/acsomega.4c02670 PMC11223217

