# Supplementary material for: Cyclooctyne End‐Functionalized Poly(morpholine‐2,5‐dione)s
Source: Macromol Rapid Commun. 2024 Nov 29;46(3):2400705. doi: 10.1002/marc.202400705 (PMC11800061; doi:10.1002/marc.202400705)
Supplement: Supplementary file 1 — Supporting Information [file MARC-46-2400705-s001.pdf]

Macromolecular Rapid Communications

Supporting Information

for *Macromol. Rapid Commun.*, DOI 10.1002/marc.202400705

Cyclooctyne End-Functionalized Poly(morpholine-2,5-dione)s

Johanna Schreiber, Natalie E. Göppert, Leanne M. Stafast, Christine Weber and Ulrich S. Schubert**

Supporting Information

Cyclooctyne End-Functionalized Poly(morpholine-2,5-dione)s

Johanna Schreiber, Natalie E. Göppert, Leanne M. Stafast, Christine Weber, Ulrich S. Schubert**

Experimental Section

Materials

L-Phenylalanine, L-valine and L-isoleucine (> 98%) were purchased from TCI. Chloroacetyl chloride (≥ 99.0%), triethylamine (NEt_3, ≥ 99.5%), 1,8-diazabicyclo[5.4.0]undec-7-ene (DBU, *purissimum*, ≥ 99.0%), benzoic acid (≥ 99.5%) as well as (1*R*,8*S*,9*S*)-bicyclo-[6.1.0]non-4-yn-9-ylmethanol (BCN-OH) were supplied by Sigma-Aldrich. 1-(3,5-Bis(trifluoromethyl)phenyl)-3-cyclohexylthiourea (TU) was synthesized following a procedure of Göppert *et al.*[1] The synthesis of **Ret-PEtOx-N_3** is described by Stafast *et al.*[2] Solvents for synthesis were dried in a solvent purification system (Pure solv EN, InnovativeTechnology). All other chemicals were obtained from the usual suppliers and, unless otherwise mentioned, used without further purification.

Instrumentation

The homopolymer syntheses were carried out under a nitrogen atmosphere in a UNIlab Plus glovebox from MBRAUN, equipped with a vacuum pump, a UNIlab SP gas purification system and MB B-L-03 Hepa filters.

^{1}H NMR spectra were measured in CD_2Cl_2, $CDCl_3$ or deuterated dimethyl sulfoxide (DMSOd_6) at 298 K using a 300 MHz Avance I spectrometer from Bruker, equipped with a dual proton (^{1}H) and carbon (^{13}C) sample head and an automatic BACS 120 sample changer (Bruker, Germany).

Diffusion-ordered NMR spectroscopy (DOSY NMR) was measured with an Avance NEO 500 spectrometer (Bruker), equipped with a CPP BBFO Prodigy probehead and an automatic sample loading system (SampleCasePlus) for high-throughput sample analysis. All chemical shifts (δ) are given in ppm in reference to the residual solvent signal.

Size exclusion chromatography (SEC) was measured on an Agilent 1200 series system, equipped with a PSS degasser, a G1310A pump, a G1329A auto sampler and a Techlab oven (40 °C). A G7162A refractive index detector (RID) as well as a G1315D diode array detector (DAD) was utilized for data acquisition. A solution of 0.21% w/w LiCl in *N*,*N*-dimethylacetamide (DMAc) was used as eluent. The flow rate was adjusted to 1 mL min^{-1}. A PSS Gram guard 30 Å and a PSS Gram guard 1,000 Å column were placed in series and served as a column set (10 µm particle size). The number averages of the molar mass (M_n) were

determined using poly(styrene) (PS, Agilent Technologies, 400 to 1,000,000 g mol^{-1}) as calibration standard.

Matrix-assisted laser desorption/ionization-time of flight-mass spectrometry (MALDI-TOF MS) was performed on a RapifleX MALDI-TOF/TOF system (Bruker Daltonics), equipped with a ScoutMTP II ion source and a Smartbeam™ 3D laser (355 nm wavelength). The spectra were measured in positive reflector mode using *trans*-2-[3-(4-*tert*-butylphenyl)-2-methyl-2-propenylidene]malononitrile (DCTB) or dithranol as matrix and sodium trifluoroacetate (NaTFA) as doping salt. The data were processed with the manufacturer's software FlexAnalysis 4.0. Poly(methyl methacrylate) (2,500 Da) or FleXstandard™ (Bruker) were used as calibration standards.

Monomer synthesis

The monomer syntheses were performed following a procedure published by Göppert *et al.*[1]

General procedure for the precursor synthesis:

Under argon atmosphere, 1 eq. L-amino acid was suspended in dry tetrahydrofuran (THF). 1 eq. NEt_3 was added to the reaction mixture, which was stirred for 20 min at room temperature. The mixture was cooled to −5 °C using a NaCl-ice bath. A solution of 1.2 eq. chloroacetyl chloride in dry THF was added dropwise over a period of 20 min while the temperature was kept below 0 °C. Thereby, a final concentration of 0.3 mol L^{-1} of the amino acid was reached. Stirring was continued further 30 min. Afterwards, the reaction mixture was allowed to reach room temperature and stirred for another 1.5 h.

The mixture was diluted with 100 mL distilled water and washed with 100 mL brine. The combined aqueous phases were extracted three times with 100 mL ethyl acetate. The organic phases were dried over Na_2SO_4 and filtered. Subsequently, the solvent was removed at reduced pressure. The residue was shortly suspended in diethyl ether. After the filtration of the suspension, the solvent was again removed under reduced pressure. For the following cyclization step, the crude product was used without further purification.

General procedure for the cyclization reaction:

8 eq. $NaHCO_3$ were suspended in *N*,*N*-dimethylformamide (DMF). The suspension was heated to 60 °C. Subsequently, 1 eq. of the precursor dissolved in DMF was added dropwise over a

period of 30 min to achieve a final concentration of 0.03 mol L^{-1} of the reactant. The reaction mixture was stirred overnight at 60 °C.

After filtering the mixture, the solvent was removed under reduced pressure. The residue was suspended in $CHCl_3$ to further remove DMF. The received suspension was filtered. The filtrate was dried over $MgSO_4$ and filtered. The solvent was removed at reduced pressure. Further purification was carried out by recrystallization using a 1:1 (v/v) mixture of *n*-hexane and ethyl acetate. The obtained monomers were dried *in vacuo*.

Yield of the L-phenylalanine-based PheG: 30%.

Yield of the L-isoleucine-based IleG: 18%.

Yield of the L-valine-based ValG: 25%.

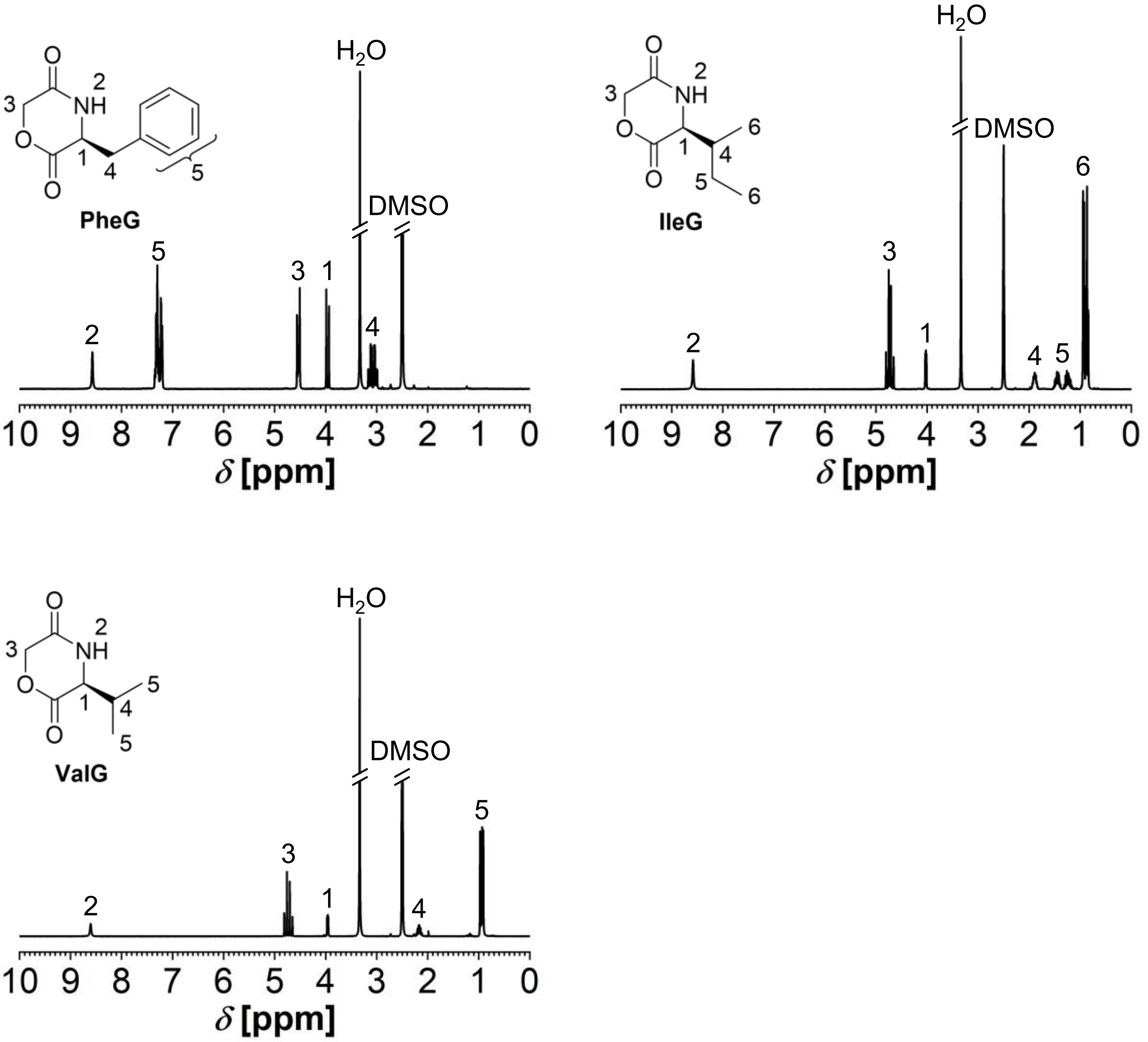

Figure S1. ^{1}H NMR spectra (300 MHz, DMSO-d_6) of the synthesized monomers and assignment of the signals to the schematic representation of the structures.

Ring-opening polymerizations

All polymerizations were carried out in a glovebox based on a procedure published by Göppert *et al.*[1] Preheated vials were used as reaction vessels, which were cooled to room temperature under argon atmosphere. The targeted [M]/[I]/[DBU]/[TU] was 100/1/1/10, if not stated otherwise.

General procedure for the performance of the kinetic studies:

Monomer and TU were dissolved in dry CH_2Cl_2 before the required volume of DBU stock solution was added. The final monomer concentration was 0.5 mol L^{-1} or 0.3 mol L^{-1}, respectively. BCN-OH was added to the reaction solution to initiate the polymerization. The reaction mixture was stirred at room temperature. Samples were taken at certain time intervals and transferred to vials containing at least 10 eq. of benzoic acid to stop the reaction. Those were investigated by ^{1}H NMR spectroscopy and SEC.

Polymerization kinetics with PheG at a concentration of 0.5 mol L^{-1}:

200.0 mg PheG (974.6 µmol), 1.49 mg BCN-OH (9.9 µmol), 1.46 µL DBU (1.49 mg; 9.8 µmol) and 36.1 mg TU (97.5 µmol) were used. The total volume of dry CH_2Cl_2 was 1.949 mL. The reached [M]/[I]/[DBU]/[TU] was 98/1/1/10.

Polymerization kinetics with PheG at a concentration of 0.3 mol L^{-1}:

117.9 mg PheG (574.5 µmol), 0.86 mg BCN-OH (5.7 µmol), 0.86 µL DBU (0.87 mg; 5.7 µmol) and 21.3 mg TU (57.5 µmol) were used in an overall volume of 1.915 mL dry CH_2Cl_2.

Polymerization kinetics with IleG at a concentration of 0.3 mol L^{-1}:

87.6 mg IleG (511.7 µmol), 0.77 mg BCN-OH (5.1 µmol), 0.76 µL DBU (0.78 mg; 5.1 µmol) and 19.0 mg TU (51.3 µmol) were used. The total volume of solvent used was 1.726 mL dry CH_2Cl_2.

Polymerization kinetics with ValG at a concentration of 0.3 mol L^{-1}:

126.4 mg ValG (804.2 µmol), 1.21 mg BCN-OH (8.0 µmol), 1.20 µL DBU (1.22 mg; 8.0 µmol) and 29.8 mg TU (80.5 µmol) were used in an overall volume of 2.678 mL dry CH_2Cl_2.

General polymerization procedure:

Monomer and TU were dissolved in a specific volume of dry CH_2Cl_2 to achieve a monomer concentration of 0.3 mol L^{-1}, taking into account the required amount of DBU stock solution. The corresponding volume of the latter was added afterwards. Then, the reaction was stirred at room temperature. The reaction times and thus the conversions were selected individually based on the results of the kinetic studies. The polymerizations were stopped by adding 10 eq. benzoic acid. An aliquot was taken to determine the monomer conversion by means of ^1H NMR spectroscopy. For purification, the polymers were precipitated several times in cold diethyl ether (−80 °C) and dried *in vacuo*.

Synthesis of **BCN-PPheG**:

1.182 g PheG (5.758 mmol), 8.65 mg BCN-OH (57.6 µmol), 9.46 µL DBU (9.65 mg; 63.4 µmol) and 213.3 mg TU (575.9 µmol) were used in an overall volume of 19.19 mL dry CH_2Cl_2. 70 mg Benzoic acid (573.2 µmol) were used at a reaction time of 45 min to stop the polymerization.

Yield: 103 mg. Conversion: 29%. DP_{theo}: 29.

^1H NMR (300 MHz, CD_2Cl_2): δ = 3.02-3.30 (m, 2 H, CH_2, substituent); 4.06-4.75 (m, 3 H, CH, CH_2, backbone); 7.04-7.39 (m, 5 H, aryl); 7.62-7.92 (m, 1 H, NH) ppm.

SEC (DMAc, 0.21% w/w LiCl; RID; PS calibration): M_n = 20,600 g mol^{-1}; Đ = 1.19.

MALDI-TOF MS (DCTB, NaTFA): $[C_{10}H_{13}O(C_{11}H_{11}NO_3)_nH+Na]^+$, $[C_{10}H_{13}O(C_{11}H_{11}NO_3)_nH+K]^+$, $[HO(C_{11}H_{11}NO_3)_nH+Na]^+$, $[NaO(C_{11}H_{11}NO_3)_nH+Na]^+$ and $[(C_{11}H_{11}NO_3)_n+Na]^+$ observed.

Synthesis of **BCN-PIleG**:

409.9 mg IleG (2.394 mmol), 3.60 mg BCN-OH (23.9 µmol), 3.57 µL DBU (3.64 mg; 23.9 µmol) and 88.8 mg TU (239.8 µmol) were used in an overall volume of 7.984 mL dry CH_2Cl_2. 29.3 mg Benzoic acid (239.9 µmol) were used at a reaction time of 60 min to stop the reaction.

Yield: 139 mg. Conversion: 81%. DP_{theo}: 81.

^1H NMR (300 MHz, $CDCl_3$): δ = 0.66-1.11 (m, 6 H, CH_3, substituent); 1.12-1.65 (m, 2 H, CH_2, substituent); 1.92-2.18 (m, 1 H, CH, substituent); 4.06-4.99 (m, 3 H, CH, CH_2, backbone); 7.37-8.00 (m, 1 H, NH) ppm.

SEC (DMAc, 0.21% w/w LiCl; RID; PS calibration): M_n = 25,800 g mol^{-1}; Đ = 1.25.

MALDI-TOF MS (Dithranol, NaTFA): $[C_{10}H_{13}O(C_8H_{13}NO_3)_nH+Na]^+$, $[HO(C_8H_{13}NO_3)_nH+Na]^+$ and $[NaO(C_8H_{13}NO_3)_nH+Na]^+$ observed.

Synthesis of **BCN-PValG**:
1.282 g ValG (8.156 mmol), 12.25 mg BCN-OH (81.6 µmol), 12.18 µL DBU (12.42 mg; 81.6 µmol) and 302.1 mg TU (815.7 µmol) were used in an overall volume of 27.189 mL dry CH_2Cl_2. 100.0 mg Benzoic acid (818.8 µmol) were used at a reaction time of 120 min to stop the reaction.
Yield: 1.047 g. Conversion: 83%. DP_{theo}: 83.
^{1}H NMR (300 MHz, $CDCl_3$): δ = 0.79-1.12 (m, 6 H, CH_3, substituent); 2.19-2.40 (m, 1 H, CH, substituent); 4.05-4.96 (m, 3 H, CH, CH_2, backbone); 7.50-7.77 (m, 1 H, NH) ppm.
SEC (DMAc, 0.21% w/w LiCl; RID; PS calibration): M_n = 35,800 g mol^{-1}; Đ = 1.19.
MALDI-TOF MS (DCTB, NaTFA): $[C_{10}H_{13}O(C_7H_{11}NO_3)_nH+Na]^+$ observed.

Click reaction *via* SPAAC
For the synthesis of **Ret-PEtOx-*b*-PValG** 34.8 mg **Ret-PEtOx-N$_3$** (14.9 µmol) and 197.2 mg **BCN-PValG** (14.9 µmol) were dissolved in 2.868 mL anhydrous CH_2Cl_2. The reaction mixture was stirred for 24 h in a brown flask. The obtained polymer was precipitated in cold diethyl ether (−80 °C) and dried *in vacuo*.
Yield: 220 mg.
^{1}H NMR (300 MHz, $CDCl_3$): δ = 0.71-1.40 (m, 566 H, CH_3, PEtOx ethyl side chain/PValG *iso*-propyl side chain); 1.42-2.11 (m, 56 H, cyclohexenyl moiety, water); 2.14-2.54 (m, 123 H, CH *iso*-propyl/CH_2 ethyl side chain); 3.28-3.66 (m, 77 H, PEtOx backbone); 4.05-5.04 (m, 249 H, CH, CH_2, PValG backbone); 7.50-7.83 (m, 77 H, NH) ppm.
SEC (DMAc, 0.21% w/w LiCl; RID; PS calibration): M_n = 35,800 g mol^{-1}; Đ = 1.23.

Kinetic studies

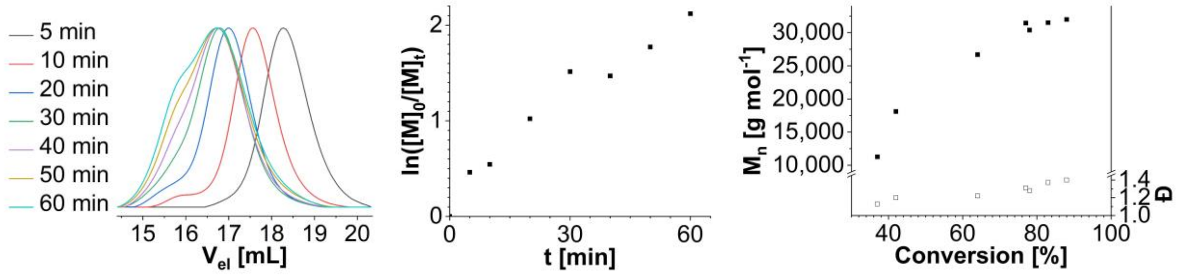

Figure S2. Overview of the results of the kinetic studies conducted for the BCN-OH-initiated ROP of PheG ([M]/[I]/[DBU]/[TU] = 98/1/1/10, $[M]_0 = 0.5$ mol L^{-1}, room temperature). Left: SEC elugrams (DMAc, 0.21% w/w LiCl; RID). Middle: Semilogarithmic kinetic plot. Right: Plot of M_n and Đ determined by SEC against the monomer conversion.

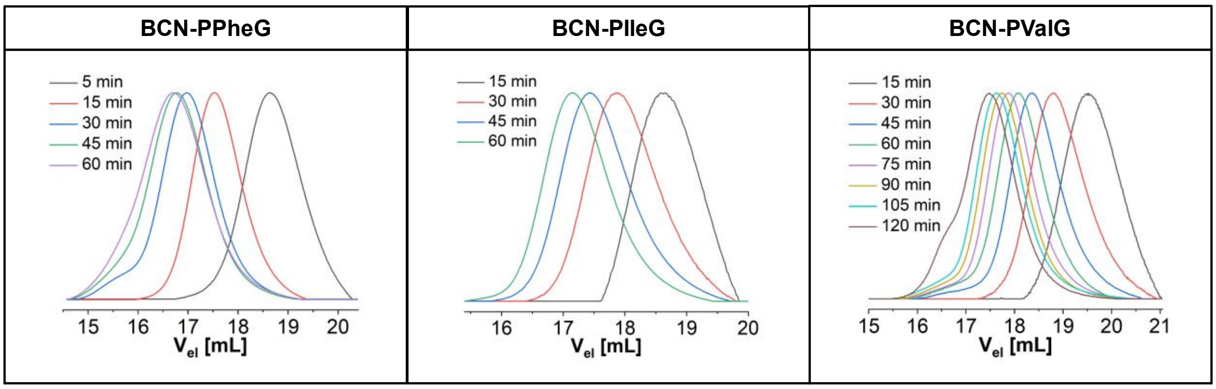

Figure S3. SEC elugrams (DMAc, 0.21% w/w LiCl; RID) obtained during the kinetic studies of the BCN-OH-initiated ROP of PheG, IleG and ValG ([M]/[I]/[DBU]/[TU] = 100/1/1/10, $[M]_0 = 0.3$ mol L^{-1}, room temperature).

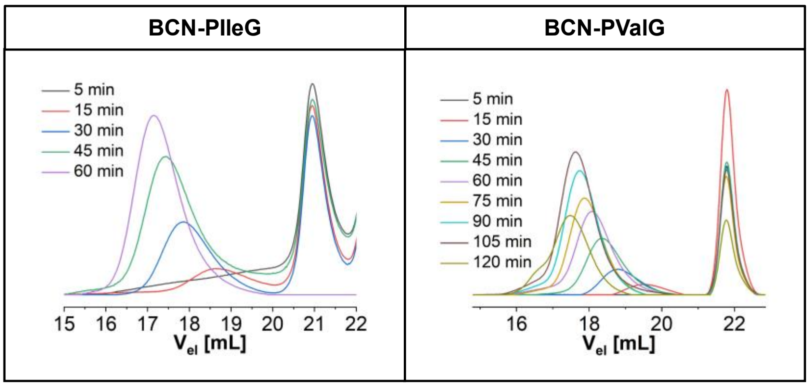

Figure S4. Overlay of the SEC elugrams (DMAc, 0.21% w/w LiCl; RID) obtained during the kinetic studies of the BCN-OH-initiated ROP ([M]/[I]/[DBU]/[TU] = 100/1/1/10, $[M]_0 = 0.3$ mol L^{-1}, room temperature) of IleG and ValG with the signals of the polymer chains at $V_{el} \leq 20$ mL and other signals of the reaction mixture at $V_{el} = 21$ mL.

Polymer characterization

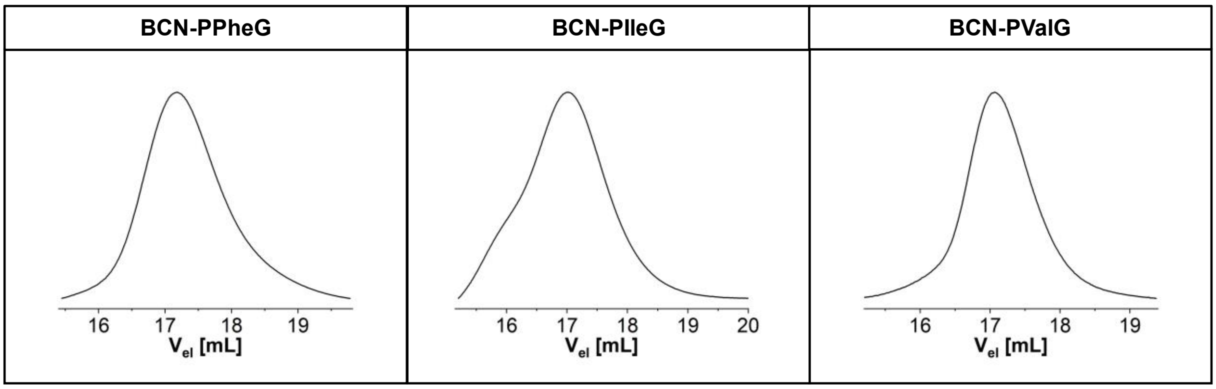

Figure S5. Overlay of the SEC elugrams (DMAc, 0.21% w/w LiCl; RID) of **BCN-PPheG**, **BCN-PIleG** and **BCN-PValG**.

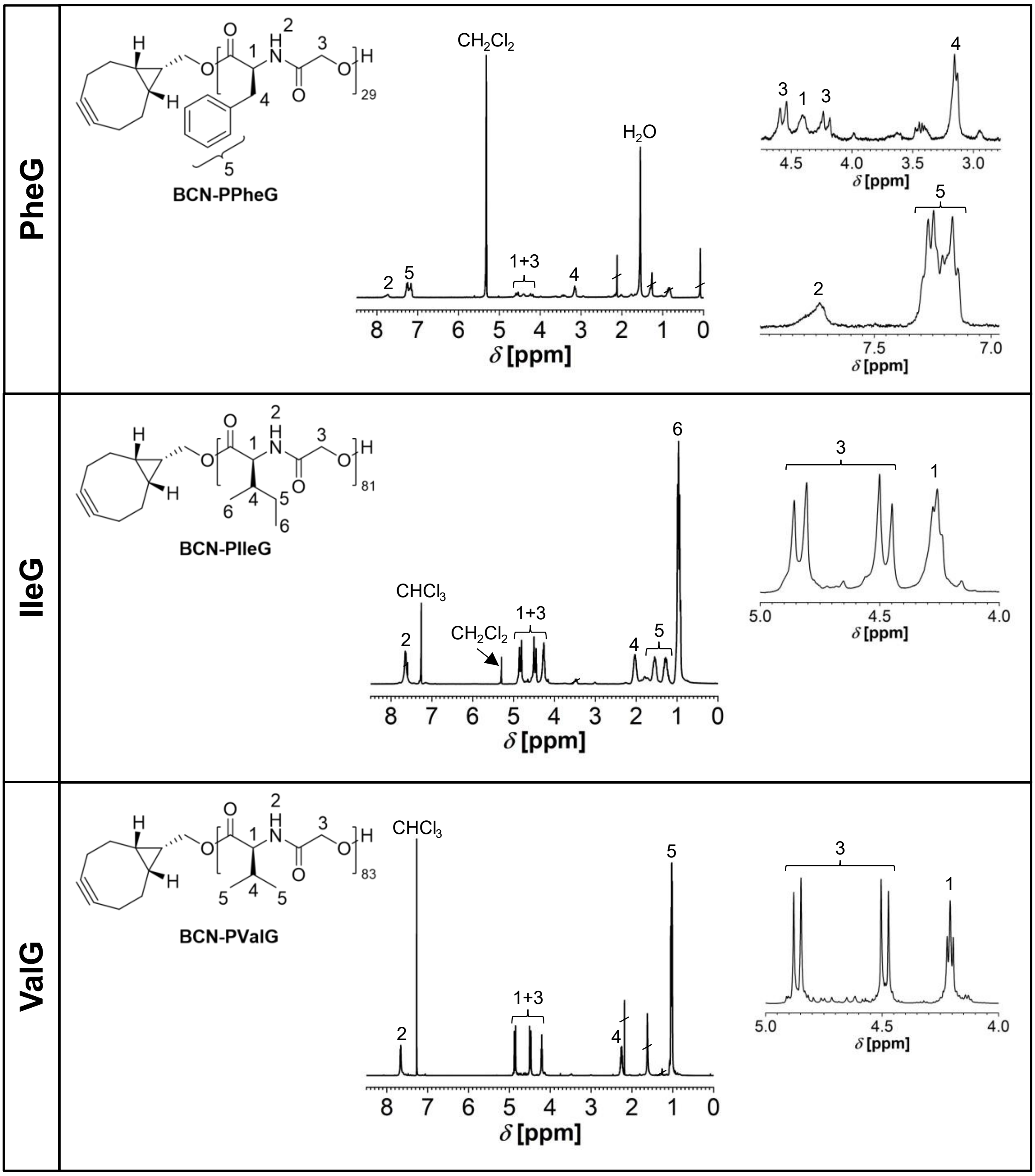

Figure S6. Top: ^{1}H NMR spectrum (300 MHz, CD_2Cl_2) of **BCN-PPheG**. Middle: ^{1}H NMR spectrum (300 MHz, $CDCl_3$) of **BCN-PIleG**. Bottom: ^{1}H NMR spectrum (300 MHz, $CDCl_3$) of **BCN-PValG**.

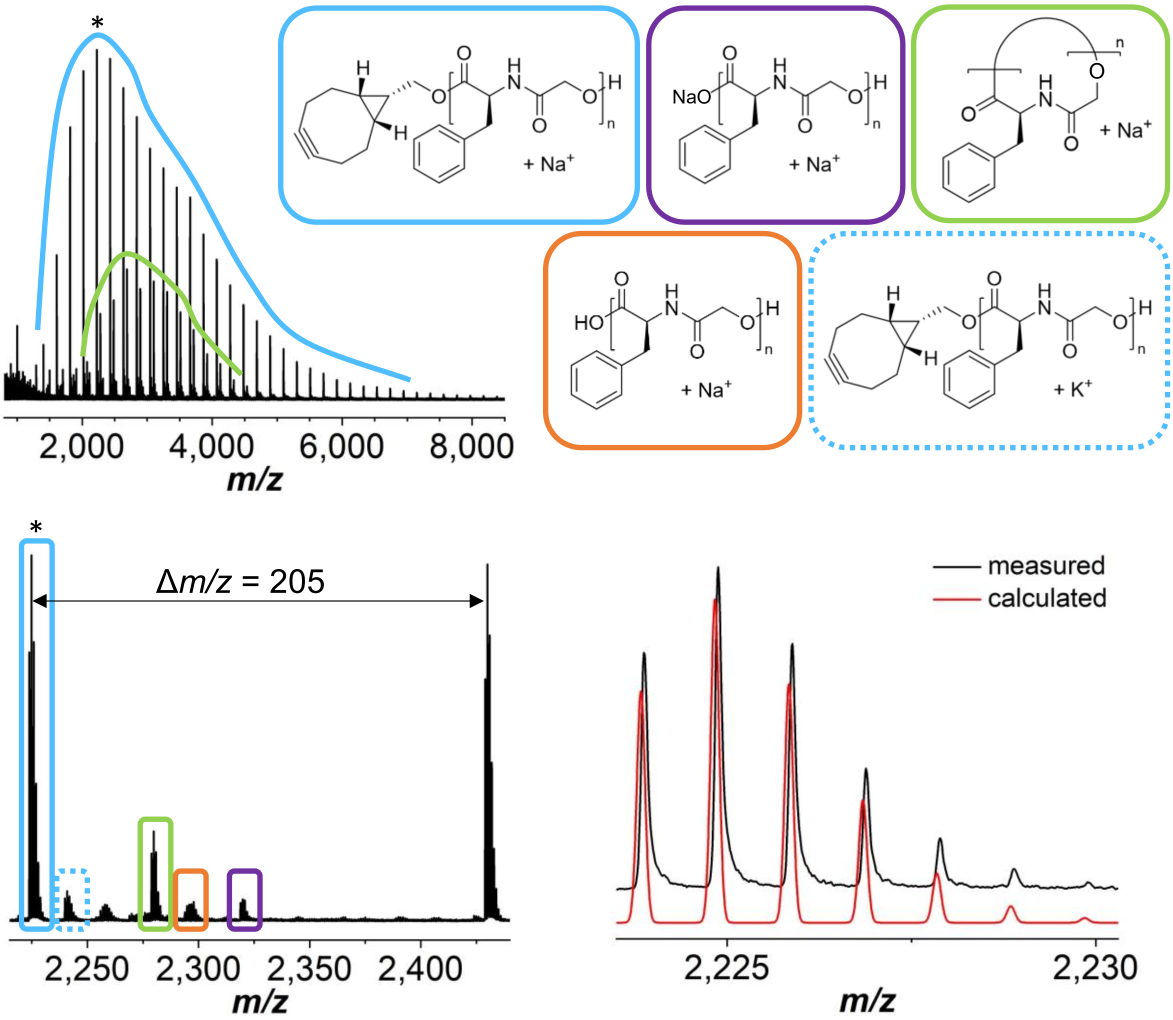

Figure S7. MALDI-TOF MS analysis of **BCN-PPheG** (DCTB, NaTFA). Top right: Summary of the detected species. Top left: Full mass spectrum of **BCN-PPheG** with highlighted distributions of the species detected with the highest intensity. Bottom left: Zoom into the spectrum. Bottom right: Overlay of the measured and the calculated isotopic pattern of the most abundant species.

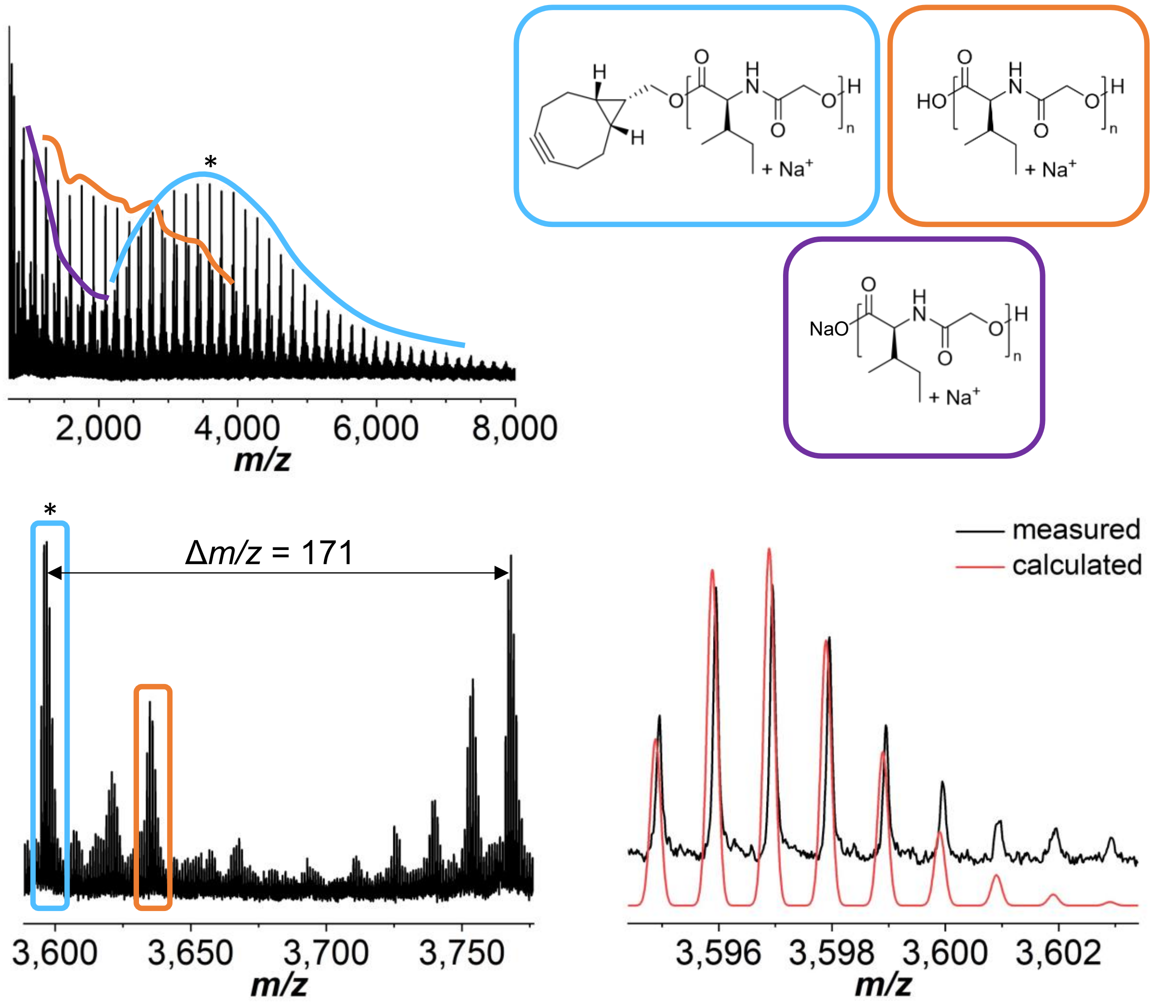

Figure S8. MALDI-TOF MS analysis of **BCN-PIleG**. Top right: Summary of the detected species. Top left: Full mass spectrum (dithranol, NaTFA) of **BCN-PIleG** with highlighted distributions of the detected species. Bottom left: Zoom into the spectrum. Bottom right: Overlay of the measured and the calculated isotopic pattern of the most abundant species.

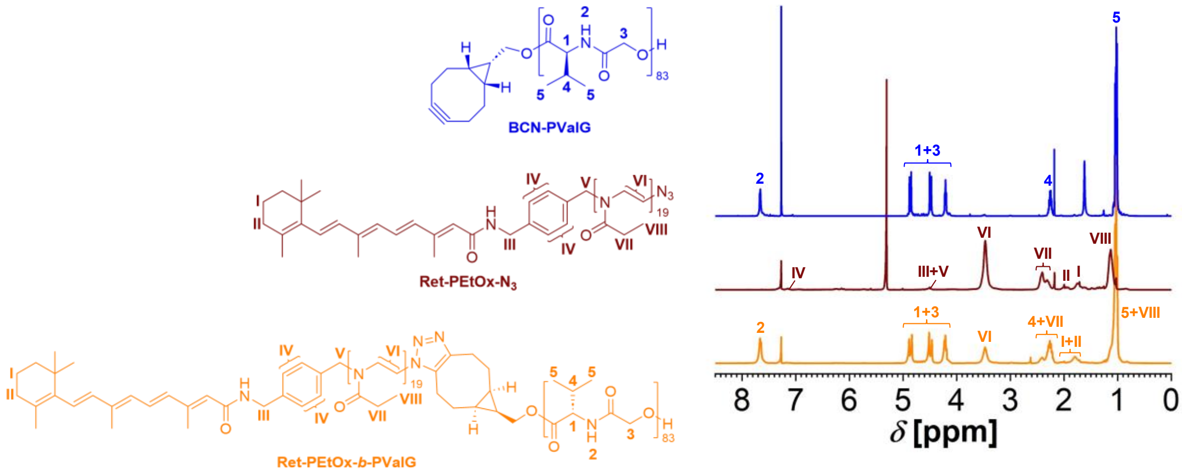

Figure S9. Overlay of the ^{1}H NMR spectra (300 MHz, $CDCl_3$) of **BCN-PValG**, **Ret-PEtOx-N$_3$** and **Ret-PEtOx-*b*-PValG**.

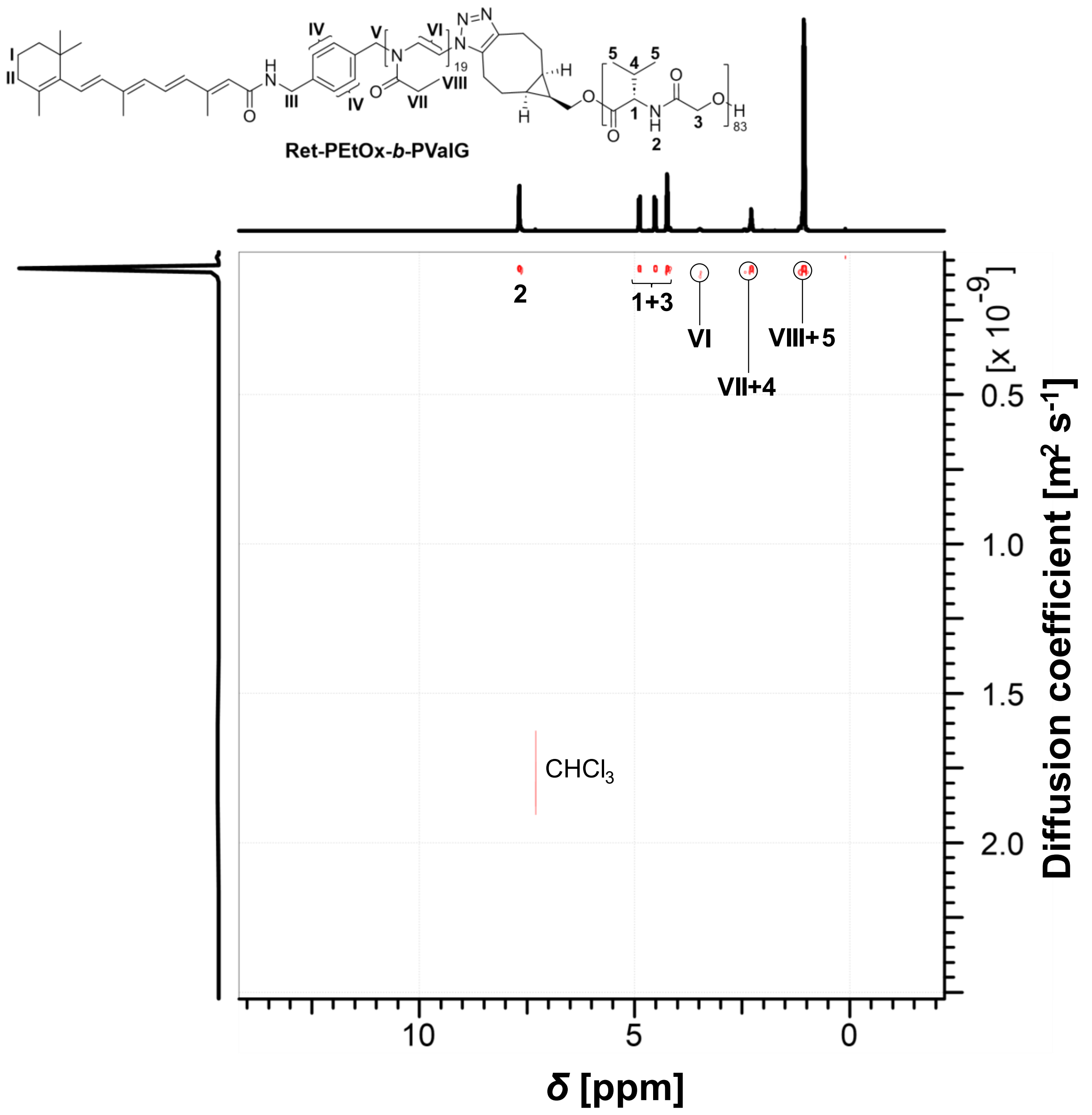

Figure S10. DOSY NMR spectrum (500 MHz, $CDCl_3$) of **Ret-PEtOx-*b*-PValG**.

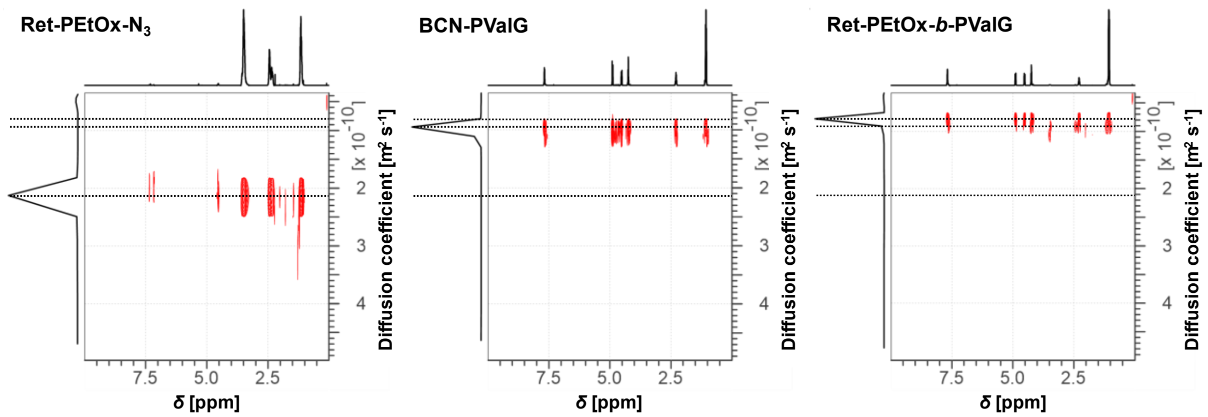

Figure S11. Overview of the zoomed in DOSY NMR spectra (500 MHz, $CDCl_3$) of **Ret-PEtOx-*b*-PValG** and the two building blocks **BCN-PValG** and **Ret-PEtOx-N_3**.

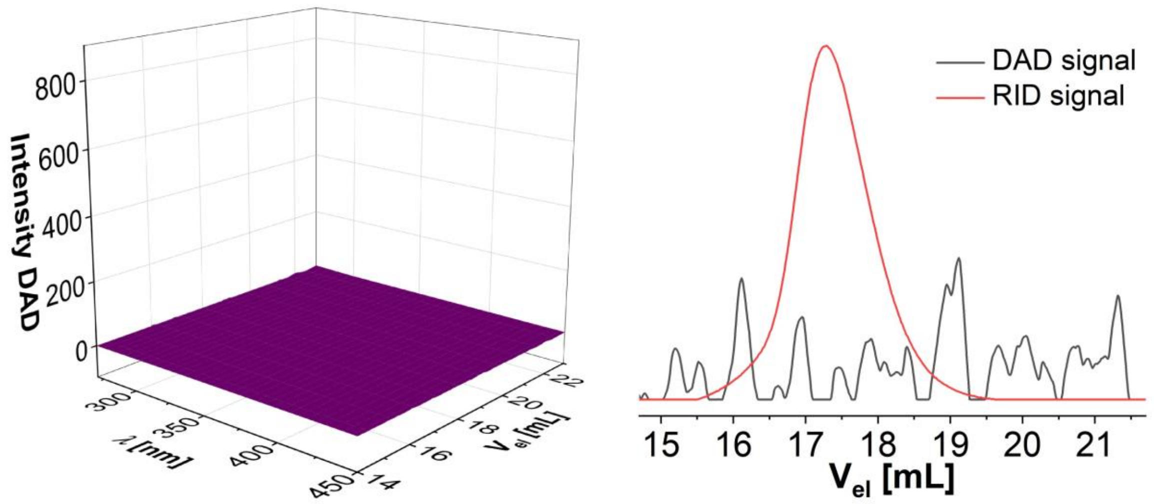

Figure S12. Left: 3D spectra of the SEC measurement (DMAc, 0.21% w/w LiCl; RID; DAD) of **BCN-PValG** using DAD. Right: Overlay of the elugrams of **BCN-PValG** recorded with the RID (red) and the DAD (351 nm, grey).

References

[1] N. E. Göppert, M. Dirauf, P. Liebing, C. Weber, U. S. Schubert, *Macromol. Rapid Commun.* **2022**, *44*, e2200651.

[2] L. M. Stafast, N. Engel, H. Görls, C. Weber, U. S. Schubert, *Eur. Polym. J.* **2023**, *184*, 111779.
